# Key pillars of active aging in the context of the Decade of Healthy Ageing (2021–2030): an integrative literature review

**DOI:** 10.1590/1980-5764-DN-2025-0439

**Published:** 2026-08-24

**Authors:** Michel Siqueira da Silva, Ana Laís Gomes da Silva, Fátima Heloyse Alexandria Firmino da Silva, Maria Caroline Nascimento Pereira da Luz, Vilani Medeiros de Araújo Nunes, Gilson de Vasconcelos Torres

**Affiliations:** 1 Universidade Federal do Rio Grande do Norte, Programa de Pós-Graduação em Ciências da Saúde, Natal RN, Brazil. Universidade Federal do Rio Grande do Norte Programa de Pós-Graduação em Ciências da Saúde RN Natal Brazil; 2 Universidade Federal do Rio Grande do Norte, Departamento de Saúde Coletiva, Natal RN, Brazil. Universidade Federal do Rio de Janeiro Universidade Federal do Rio Grande do Norte Departamento de Saúde Coletiva RN Natal Brazil

**Keywords:** Healthy aging, Healthy lifestyles, Aged, Aging, Envelhecimento saudável, Estilo de vida saudável, Idoso, Envelhecimento

## Abstract

The number of older adults worldwide has increased significantly, and by 2030 one in six people will be aged 60 years or older. Population aging is a central issue in global health agendas. The Decade of Healthy Ageing (2021–2030) aims to improve the quality of life of older adults.

**Objective:**

To identify key pillars associated with active aging within this framework.

**Methods:**

Integrative literature review conducted in six stages using the Population-Concept-Context (PCC) strategy. Searches were performed in the United States National Library of Medicine (PubMed), Web of Science, Scopus, and Scientific Electronic Library Online (SciELO), including studies published between 2020 and 2025.

**Results:**

Five dimensions were identified: healthy lifestyle behaviors, social engagement and cognitive stimulation, spirituality, socioeconomic determinants, and digital health technologies, linked to autonomy and quality of life.

**Conclusion:**

Active aging is a multidimensional process requiring intersectoral and person-centered strategies aligned with public policies. However, the available evidence remains uneven across domains, with a predominance of studies focused on individual behavioral factors.

## RESUMO

O número de pessoas idosas no mundo tem aumentado de forma significativa, e estima-se que, até 2030, uma em cada seis pessoas terá 60 anos ou mais. Nesse contexto, o envelhecimento populacional ocupa posição central nas agendas globais de saúde. A Década do Envelhecimento Saudável (2021–2030) constitui um plano de ação global voltado à melhoria da qualidade de vida das pessoas idosas.

**Objetivo:**

Identificar os principais pilares associados ao envelhecimento ativo no contexto da Década do Envelhecimento Saudável.

**Métodos:**

Revisão integrativa da literatura, conduzida em seis etapas, com base na estratégia Population-Concept-Context (PCC). A busca foi realizada nas bases United States National Library of Medicine (PubMed), Web of Science, Scopus e Scientific Electronic Library Online (SciELO), incluindo estudos publicados entre 2020 e 2025, nos idiomas português, inglês e espanhol.

**Resultados:**

Foram identificadas cinco dimensões centrais: hábitos de vida saudáveis, engajamento social e estímulo cognitivo, espiritualidade, determinantes socioeconômicos e tecnologias digitais em saúde, dimensões associadas à autonomia, funcionalidade e qualidade de vida.

**Conclusão:**

O envelhecimento ativo é um processo multidimensional que requer estratégias intersetoriais, centradas na pessoa idosa e integradas às políticas públicas. No entanto, as evidências disponíveis ainda são desiguais entre os diferentes domínios, com predominância de estudos focados em fatores comportamentais individuais.

## INTRODUCTION

Population aging is one of the most significant demographic transformations of our time. Life expectancy has been increasing worldwide due to advances in medicine and improvements in living conditions. This demographic shift brings a series of challenges for both individuals and societies, particularly in ensuring independence for older adults^1^.

Since 2020, the Pan American Health Organization (PAHO) has led a joint agenda through interprogrammatic, interagency, and intersectoral collaboration known as the “Decade of Healthy Ageing in the Americas (2021–2030),” declared by the United Nations General Assembly in December 2020 and considered a key strategy for building a society for all ages^2^. This global initiative brings together the efforts of governments, civil society, international agencies, professional teams, academia, the media, and the private sector to improve the lives of older people, their families, and their communities.

This initiative seeks to create opportunities for collaborative action and to improve functional ability by 2030, ensuring the meaningful participation and empowerment of older people throughout all stages of the process^1^. It is organized around four action areas articulated across different levels and sectors:

- Changing the way we think, feel, and act toward age and aging;
- Ensuring that communities foster the abilities of older people;
- Delivering integrated care and primary health care services that are person-centered and responsive to the needs of older adults; and
- Providing access to long-term care for older people who require it.

Healthy aging involves creating opportunities for individuals to achieve what they value throughout their lives. In this context, the principles of the Decade of Healthy Ageing in the Americas (2021–2030) emphasize optimizing functional ability, which results from the interaction between intrinsic capacity and the environment. Functional ability includes meeting basic needs, maintaining autonomy, mobility, relationships, and social participation. Intrinsic capacity encompasses physical, mental, and psychological functions such as locomotion, vision, hearing, vitality, and cognition. Environments, in turn, include homes, communities, societies, services, and policies that influence how these capacities can be expressed and sustained^1,2^.

In this study, healthy ageing is understood as the process of developing and maintaining the functional ability that enables well-being in older age, whereas active ageing refers to the optimization of opportunities for health, participation, and security in order to enhance quality of life as people age, in accordance with the definitions proposed by the World Health Organization (WHO).

Although the concept of healthy ageing has been widely adopted in global health discourse, it has been criticized for placing excessive emphasis on individual responsibility while underrepresenting structural determinants such as inequality, access to care, and social protection systems.

Considering that longevity depends on both individual conditions and social and environmental contexts throughout the life course, the present study sought to answer the following question: which factors associated with healthy aging have been identified in the scientific literature, and how do these factors relate to the guidelines of the Decade of Healthy Ageing (2021–2030)?

### General objective

To analyze, in the scientific literature, the main factors associated with healthy aging, to discuss their relationship with the guidelines of the Decade of Healthy Ageing (2021–2030), and to critically examine how these factors reflect or diverge from the strategic priorities of the Decade of Healthy Ageing.

### Specific objectives

- To characterize the included studies according to their main methodological and thematic characteristics.
- To identify the factors associated with healthy aging described in the selected studies.
- To analyze how the findings reported in the literature relate to the guidelines of the Decade of Healthy Ageing, proposed by WHO.

## METHODS

This study consists of an integrative literature review aimed at synthesizing results derived from scientific evidence within the context of the Decade of Healthy Ageing. The review was conducted following six methodological stages:

- Formulation of the guiding research question;
- Literature search and sampling;
- Data collection;
- Critical analysis of the included studies;
- Discussion of the results; and
- Presentation of the integrative review^3^.

The guiding research question was: *Which factors associated with healthy aging have been identified in the scientific literature, and how do these factors relate to the guidelines of the Decade of Healthy Ageing (2021–2030)?*

The research question and search strategy were structured using the Population-Concept-Context (PCC) framework, recommended by the Joanna Briggs Institute (JBI) to objectively define the research problem and guide the evidence search. The PCC model comprises: P (population), referring to the group under investigation; C (concept), corresponding to the central phenomenon or theme of the study; and C (context), which defines the setting in which the phenomenon occurs (Peters et al., 2015). In this study, the following elements were defined^4^:

- Population: studies involving individuals aged 60 years or older.
- Concept: research investigating determinants, strategies, or indicators related to healthy aging.
- Context: scientific publications published between 2020 and 2025, aligned with the guidelines established by the Decade of Healthy Ageing (2021–2030).

The complete search strategies used in each database, including Boolean operators, applied filters, and the number of records retrieved before and after filtering, are presented in Supplementary Material Table S1 (available at https://www.demneuropsy.org/wp-content/uploads/2026/05/DN-2025.0439-Supplementary-Material.docx).

The search for scientific evidence was conducted in the following databases: the United States National Library of Medicine, National Institutes of Health (PubMed), Web of Science (Clarivate Analytics), Elsevier (Scopus), and Scientific Electronic Library Online (SciELO).

Descriptors and free terms related to healthy aging and health-promoting behaviors were combined using the Boolean operators AND OR.

Studies published between 2020 and 2025, available in full text and written in Portuguese, English, or Spanish, addressing factors related to the promotion of healthy aging or active aging, were included. Editorials, letters to the editor, commentaries, duplicate studies, and publications that did not address the research question were excluded.

Study screening was conducted using the Rayyan platform, a tool widely used for managing systematic reviews. The software was employed to organize references, identify and remove duplicates, and support the screening process. Study selection was performed by two independent reviewers who assessed titles and abstracts. In cases of disagreement, studies were reassessed through consensus.

### Methodological quality assessment

The methodological quality of the included studies was assessed using the JBI Critical Appraisal Tools, according to the specific design of each study. Appropriate checklists were applied for cross-sectional studies, cohort studies, randomized controlled trials, qualitative studies, and review-based evidence, when applicable. Two reviewers independently conducted the appraisal. Disagreements were resolved through discussion and consensus. The results of the methodological quality assessment were considered in the interpretation of the findings, particularly regarding the strength, consistency, and limitations of the available evidence.

After the eligibility stage, the included articles were analyzed in full, and relevant data were extracted using an instrument previously developed by the authors to systematize the information. This instrument was structured as an electronic spreadsheet (Microsoft Excel), allowing standardized organization of the extracted data, including author, year of publication, study objective, methodological design, and main results.

### Data analysis and thematic synthesis

The extracted data were analyzed using an integrative thematic approach. First, the main findings reported in each study were subjected to initial coding according to their relevance to healthy ageing. Second, codes with conceptual similarity were grouped into preliminary thematic categories. Third, these categories were iteratively compared across the included studies to identify patterns, convergences, and complementary dimensions. Fourth, the resulting categories were refined and validated through discussion between the reviewers, considering both the recurrence of themes in the literature and their analytical alignment with the framework of the Decade of Healthy Ageing (2021–2030). This process led to the identification of five thematic dimensions: healthy lifestyle behaviors, social and cognitive engagement, spirituality, socioeconomic determinants, and digital health technologies.

The findings were interpreted in light of the guidelines established by the Decade of Healthy Ageing, allowing the identification of convergences between the scientific literature and the dimensions proposed by this global initiative. The protocol of this review was registered on the Open Science Framework (OSF) platform under DOI 10.17605/OSF.IO/AM3HB and is publicly available^5^.

**Figure 1:**
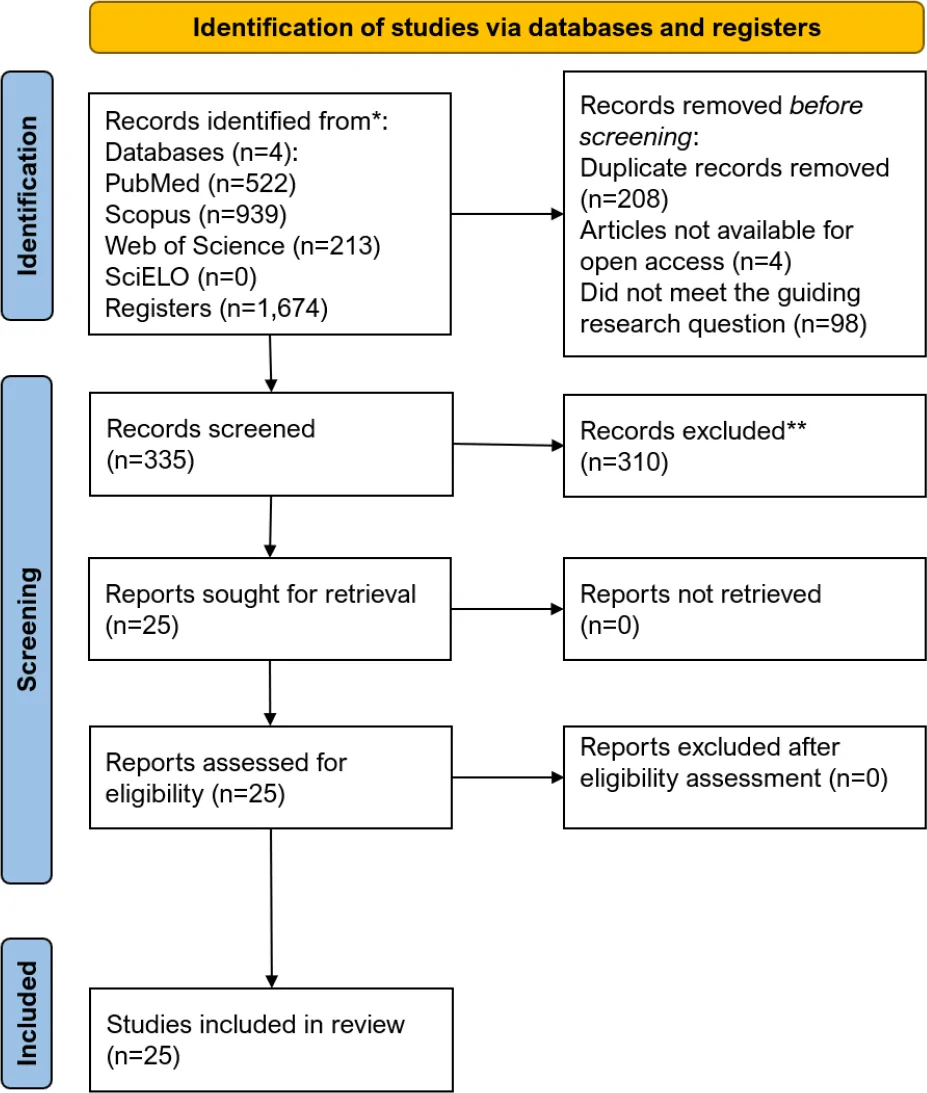
Flowchart of the article selection process (Preferred Reporting Items for Systematic Reviews and Meta-Analyses — PRISMA).

Source: Adapted and translated from Page et al.^6^

The selection process is detailed in Figure 1, adapted from the Preferred Reporting Items for Systematic Reviews and Meta-Analyses (PRISMA)^6^ model.

## RESULTS

Twenty-five studies published between 2020 and 2025 were included, originating from different countries and sociocultural contexts, and addressing multiple dimensions related to healthy aging. The studies presented methodological diversity, including cross-sectional research, cohort studies, clinical trials, literature reviews, and qualitative investigations. This methodological heterogeneity is expected in integrative reviews, as this type of synthesis seeks to gather different forms of scientific evidence in order to broaden the understanding of complex phenomena related to health and aging.

The 25 included studies were conducted in diverse geographical settings, including Asia, Europe, North America, and Latin America, reflecting a broad range of sociocultural contexts and health systems. The studies also showed considerable variation in sample size and methodological scope, ranging from small qualitative and pilot studies to large population-based cohorts and randomized controlled trials. This diversity broadened the analytical scope of the review, while also introducing heterogeneity in the type and strength of the available evidence.

The methodological quality of the included studies varied according to study design. Overall, cohort studies and randomized controlled trials demonstrated greater methodological robustness, particularly regarding control of confounding factors and longitudinal follow-up. In contrast, cross-sectional studies more frequently presented limitations related to confounding control, measurement bias, and causal inference. Detailed appraisal results are presented in Supplementary Material Table S2 (available at https://www.demneuropsy.org/wp-content/uploads/2026/05/DN-2025.0439-Supplementary-Material.docx).

Initially, the included studies were characterized according to author, year of publication, population mentioned in the title, and main thematic focus. This step aimed to identify the overall landscape of the analyzed literature and to understand which dimensions of healthy aging have been most frequently investigated (Table 1)^7,8,9,10,11,12,13,14,15,16,17,18,19,20,21,22,23,24,25,26,27,28,29,30,31^.

**Table 1:** Characterization of the studies included in the integrative review (2020–2025).

No.	Author	Study title	Population mentioned in the title	Main theme	Methodological quality	Level of evidence
1	Alali et al.^7^	Awareness of healthy lifestyle among elderly population during aging in Al-Ahsa, Saudi Arabia	Elderly population	Healthy lifestyle awareness	Moderate	Low
2	Weiner-Light et al.^8^	The role of spirituality in conceptualizations of health maintenance and healthy aging among Latin American immigrants	Latin American immigrants	Spirituality and healthy aging	Moderate	Moderate
3	Andreo-López et al.^9^	Influence of the Mediterranean diet on healthy aging	Not specified in the title	Healthy diet and aging	Moderate	Low
4	Cacciata et al.^10^	Digital health technologies to promote healthy eating and physical activity and reduce risk factors for cardiovascular disease in older adults: a pilot study	Older adults	Digital health technologies	Moderate	Moderate
5	Parrott et al.^11^	The association between dietary pattern adherence, cognitive stimulating lifestyle, and cognitive function among older adults	Older adults from the Quebec Longitudinal Study on Nutrition and Successful Aging	Diet, cognitive stimulation, and	Moderate	Low
6	Eymundsdottir et al.^12^	Lifestyle and 25-hydroxy-vitamin D among community-dwelling old adults with dementia, mild cognitive impairment, or normal cognitive function	Community-dwelling old adults	Lifestyle and vitamin D	Moderate	Low
7	Martin et al.^13^	Healthy aging among centenarians and near-centenarians: findings from the Georgia Centenarian Study	Centenarians and nearcentenarians	Healthy aging determinants	Moderate	Low
8	Dhana et al.^14^	Healthy lifestyle and life expectancy with and without Alzheimer’s dementia: population based cohort study	Not specified in the title	Lifestyle and life expectancy	High	High
9	Lee-Bravatti et al.^15^	Lifestyle behavioral factors and integrative successful aging among Puerto Ricans living in the mainland United States	Puerto Ricans living in the mainland United States	Multidimensional successful aging	High	High
10	Blotenberg et al.^16^	Factors associated with a healthy diet and willingness to change dietary behavior in older adults at increased risk of dementia	Older adults at increased risk of dementia	Healthy diet	Moderate	Low
11	Zadworna et al.^17^	Pathways to healthy aging: exploring the determinants of self-rated health in older adults	Older adults	Self-rated health and healthy aging	Moderate	Low
12	Zhang et al.^18^	Enhancing understanding of healthy aging based on time-varying dependencies among multidimensional health, life satisfaction, and health behaviors of older adults aged 60 years and over	Older adults aged 60 years and over	Multidimensional determinants of healthy aging	High	High
13	Lallukka et al.^19^	Joint trajectories of physical activity, health, and income before and after statutory retirement: a 22-year follow-up	Not specified in the title	Physical activity and socioeconomic determinants	High	High
14	Yoneda et al.^20^	The importance of engaging in physical activity in older adulthood for transitions between cognitive status categories and death: a coordinated analysis of 14 longitudinal studies	Older adulthood	Physical activity and cognition	High	High
15	Tiraphat et al.^21^	Active aging in ASEAN countries: influences from age-friendly environments, lifestyles, and socio-demographic factors	Not specified in the title	Lifestyle and active aging	Moderate	Low
16	Liu et al.^22^	Healthy lifestyles are associated with better vitamin D status in community-dwelling older men: the Health In Men Study (HIMS)	Community-dwelling older men	Lifestyle and vitamin D	Moderate	Low
17	Chu et al.^23^	Sedentary behavior, physical activity, social participation, and loneliness among community-dwelling older adults in China	Community-dwelling older adults	Social participation and loneliness	Moderate	Low
18	Szychowska and Drygas^24^	Physical activity as a determinant of successful aging: a narrative review article.	Not specified in the title	Physical activity and successful aging	Moderate	Low
19	Tcymbal et al.^25^	Interventions simultaneously promoting social participation and physical activity in community living older adults: a systematic review	Community living older adults	Multicomponent interventions	High	High
20	Hu and Feng^26^	Association between community-based health services and health lifestyles of	Older adults	Community-based health	Moderate	Low
21	Xu et al.^27^	Intrinsic capacity and health-promoting lifestyle in older adults: a latent class analysis	Older adults	Intrinsic capacity	Moderate	Low
22	Zainab et al.^28^	Healthy ageing: assessment of health-promoting lifestyle among the elderly population in Karachi, Pakistan	Elderly population	Health-promoting lifestyle	Moderate	Low
23	Peterson et al.^29^	Healthy ageing in the far North: perspectives and prescriptions	Not specified in the title	Contextual perspectives on healthy aging	Moderate	Low
24	Jiang et al.^30^	Effects of personal and health characteristics on the intrinsic capacity of older adults in the community: a cross-sectional study using the healthy aging framework	Older adults in the community	Intrinsic capacity determinants	Moderate	Low
25	Snigdha et al.^31^	Yoga-based lifestyle intervention for healthy ageing in older adults: a two-armed, waitlist randomized controlled trial with multiple primary outcomes	Older adults	Lifestyle intervention	High	High

Source: Authors’ elaboration based on the studies included in the integrative review (2020–2025)

The analysis of Table 1 shows that most studies investigated factors related to lifestyle, particularly physical activity, healthy diet, and health-promoting behaviors. Other relevant aspects associated with healthy aging, such as spirituality, social engagement, socioeconomic determinants, and the use of digital health technologies, were also identified, although less frequently reported in the analyzed literature.

After the characterization of the studies, a comparative analysis of the main factors associated with healthy aging described in the literature was conducted. This stage aimed to identify conceptual convergences among the analyzed studies and to group the findings into analytical categories representing relevant dimensions of active ageing. Based on this integrative thematic synthesis, the identified factors were organized into five main dimensions: healthy lifestyle behaviors, social and cognitive engagement, spirituality, socioeconomic determinants, and digital health technologies.

These dimensions were not previously defined by the authors, but emerged from the analysis of the included studies, representing recurrent or complementary themes present in the scientific literature on healthy aging. Table 2^7,8,9,10,11,12,13,14,15,16,17,18,19,20,21,22,23,24,25,26,27,28,29,30,31^ presents the convergence between the analyzed studies and the dimensions associated with active ageing.

**Table 2:** Convergence between the included studies and the dimensions associated with active ageing.

Article	Author	Study title	Main factors addressed	Associated dimension
1	Alali et al.^7^	Awareness of healthy lifestyle among elderly population during aging in Al-Ahsa, Saudi Arabia	Knowledge about healthy lifestyle	Healthy lifestyle behaviors
2	Weiner-Light et al.^8^	The role of spirituality in conceptualizations of health maintenance and healthy aging among Latin American immigrants	Spirituality and health maintenance	Spirituality
3	Andreo-López et al.^9^	Influence of the Mediterranean diet on healthy aging	Mediterranean diet and healthy aging	Healthy lifestyle behaviors
4	Cacciata et al.^10^	Digital health technologies to promote healthy eating and physical activity and reduce risk factors for cardiovascular disease in older adults: a pilot study	Use of digital technologies for health promotion	Digital health technologies
5	Parrott et al.^11^	The association between dietary pattern adherence, cognitive stimulating lifestyle, and cognitive function among older adults from the Quebec Longitudinal Study on Nutrition and Successful Aging	Cognitively stimulating lifestyle	Healthy lifestyle behaviors
6	Eymundsdottir et al.^12^	Lifestyle and 25-hydroxy-vitamin D among community-dwelling old adults with dementia, mild cognitive impairment, or normal cognitive function	Lifestyle and vitamin D	Healthy lifestyle behaviors
7	Martin and Poon^13^	Healthy aging among centenarians and near-centenarians: findings from the Georgia Centenarian Study	Quality of life and healthy aging	Social engagement
8	Dhana et al.^14^	Healthy lifestyle and life expectancy with and without Alzheimer’s dementia: population based cohort study	Lifestyle and life expectancy	Healthy lifestyle behaviors
9	Lee-Bravatti et al.^15^	Lifestyle behavioral factors and integrative successful aging among Puerto Ricans living in the mainland United States	Healthy behaviors and frailty	Healthy lifestyle behaviors
10	Blotenberg et al.^16^	Factors associated with a healthy diet and willingness to change dietary behavior in older adults at increased risk of dementia	Healthy diet and behavioral change	Healthy lifestyle behaviors
11	Zadworna^17^	Pathways to healthy aging: exploring the determinants of self-rated health in older adults	Lifestyle and memory	Healthy lifestyle behaviors
12	Zhang et al.^18^	Enhancing understanding of healthy aging based on time-varying dependencies among multidimensional health, life satisfaction, and health behaviors of older adults aged 60 years and over	Multidimensional health and life satisfaction	Social engagement
13	Lallukka et al.^19^	Joint trajectories of physical activity, health, and income before and after statutory retirement: a 22-year follow-up	Physical activity, health, and income	Socioeconomic determinants
14	Yoneda et al.^20^	The importance of engaging in physical activity in older adulthood for transitions between cognitive status categories and death: a coordinated analysis of 14 longitudinal studies	Physical activity and cognition	Healthy lifestyle behaviors
15	Tiraphat et al.^21^	Active aging in ASEAN countries: influences from age-friendly environments, lifestyles, and socio-demographic factors	Lifestyle and chronic diseases	Healthy lifestyle behaviors
16	Liu et al.^22^	Healthy lifestyles are associated with better vitamin D status in community-dwelling older men: the Health In Men Study (HIMS)	Physical activity and cognitive decline	Healthy lifestyle behaviors
17	Chu et al.^23^	Sedentary behavior, physical activity, social participation, and loneliness among community-dwelling older adults in China	Lifestyle and vitamin D	Healthy lifestyle behaviors
18	Szychowska and Drygas^24^	Physical activity as a determinant of successful aging: a narrative review article.	Diet and cognitive stimulation	Healthy lifestyle behaviors
19	Tcymbal et al.^25^	Interventions simultaneously promoting social participation and physical activity in community living older adults: a systematic review	Physical activity and cognitive status	Healthy lifestyle behaviors
20	Hu and Feng^26^	Association between community-based health services and health lifestyles of older adults: evidence from China	Lifestyle and mental health	Healthy lifestyle behaviors
21	Xu et al.^27^	Intrinsic capacity and health-promoting lifestyle in older adults: a latent class analysis	Yoga-based intervention	Healthy lifestyle behaviors
22	Zainab et al.^28^	Healthy ageing: assessment of health-promoting lifestyle among the elderly population in Karachi, Pakistan	Lifestyle, genetics, and cognition	Healthy lifestyle behaviors
23	Peterson et al.^29^	Healthy ageing in the far North: perspectives and prescriptions	Lifestyle factors	Healthy lifestyle behaviors
24	Jiang et al.^30^	Effects of personal and health characteristics on the intrinsic capacity of older adults in the community: a cross-sectional study using the healthy aging framework	Multidomain lifestyle intervention	Healthy lifestyle behaviors
25	Snigdha et al.^31^	Yoga-based lifestyle intervention for healthy ageing in older adults: a two-armed, waitlist randomized controlled trial with multiple primary outcomes	Health-promoting lifestyle	Healthy lifestyle behaviors

Source: Authors’ elaboration based on the studies included in the integrative review (2020–2025).

The distribution of studies across the identified dimensions was uneven, with a clear predominance of research related to healthy lifestyle behaviors. In contrast, dimensions such as spirituality, socioeconomic determinants, and digital health technologies were represented by fewer studies, indicating important gaps in the literature. This imbalance suggests that the current evidence base remains more strongly oriented toward individual behavioral factors than toward broader contextual and structural determinants of healthy ageing.

Most publications were concentrated in the dimension related to healthy lifestyle behaviors, particularly physical activity, balanced diet, and health-promoting behaviors. Although this dimension showed the greatest volume of evidence, most studies were observational, especially cross-sectional and cohort designs, which limits the strength of causal interpretation. By contrast, the dimensions related to social and cognitive engagement, spirituality, socioeconomic determinants, and digital health technologies were supported by a smaller and methodologically more heterogeneous body of evidence, revealing important gaps in the literature.

The predominance of studies focusing on healthy lifestyle behaviors suggests that the scientific literature has prioritized modifiable behavioral factors as central elements for promoting healthy ageing, particularly emphasizing physical activity, the adoption of healthy dietary patterns, and the maintenance of health-promoting behaviors throughout the life course.

Considering that the present study was developed in light of the conceptual framework of the Decade of Healthy Ageing (2021–2030), the results identified in the literature were subsequently analyzed in relation to the four strategic action areas proposed by the WHO: combating ageism, developing communities that foster the abilities of older adults, delivering person-centered integrated care, and ensuring access to long-term care.

This stage aimed to verify how the included studies align with the international guidelines established for the promotion of healthy ageing according to the Decade of Healthy Ageing (2021–2030). Table 3^7,8,9,10,11,12,13,14,15,16,17,18,19,20,21,22,23,24,25,26,27,28,29,30,31^ presents the correspondence between the analyzed studies and the action areas of the Decade of Healthy Ageing.

**Table 3:** Correspondence between the included studies and the action areas of the Decade of Healthy Ageing.

Article	Author	Study title (original)	Main aspect addressed	Related action area of the Decade of Healthy Ageing
1	Alali et al.^7^	Awareness of healthy lifestyle among elderly population during aging in Al-Ahsa, Saudi Arabia	Promotion of healthy behaviors	Communities that foster the abilities of older people
2	Weiner-Light et al.^8^	The role of spirituality in conceptualizations of health maintenance and healthy aging among Latin American immigrants	Spirituality and health maintenance	Person-centered integrated care
3	Andreo-López et al.^9^	Influence of the Mediterranean diet on healthy aging	Healthy diet	Communities that foster the abilities of older people
4	Cacciata et al.^10^	Digital health technologies to promote healthy eating and physical activity and reduce risk factors for cardiovascular disease in older adults: a pilot study	Digital technologies for health promotion	Person-centered integrated care
5	Parrott et al.^11^	The association between dietary pattern adherence, cognitive stimulating lifestyle, and cognitive function among older adults from the Quebec Longitudinal Study on Nutrition and Successful Aging	Dognitively stimulating lifestyle	Communities that foster the abilities of older people
6	Eymundsdottir et al.^12^	Lifestyle and 25-hydroxy-vitamin D among community-dwelling old adults with dementia, mild cognitive impairment, or normal cognitive function	Lifestyle and cognition	Person-centered integrated care
7	Martin and Poon^13^	Healthy aging among centenarians and near-centenarians: findings from the Georgia Centenarian Study	Quality of life and healthy aging	Combating ageism
8	Dhana et al.^14^	Healthy lifestyle and life expectancy with and without Alzheimer’s dementia: population based cohort study	Lifestyle and life expectancy	Person-centered integrated care
9	Lee-Bravatti et al.^15^	Lifestyle behavioral factors and integrative successful aging among Puerto Ricans living in the mainland United States	Healthy behaviors and frailty	Person-centered integrated care
10	Blotenberg et al.^16^	Factors associated with a healthy diet and willingness to change dietary behavior in older adults at increased risk of dementia	Healthy diet	Person-centered integrated care
11	Zadworna^17^	Pathways to healthy aging: exploring the determinants of self-rated health in older adults	Lifestyle and memory	Communities that foster the abilities of older people
12	Zhang et al.^18^	Enhancing understanding of healthy aging based on time-varying dependencies among multidimensional health, life satisfaction, and health behaviors of older adults aged 60 years and over	Multidimensional health and well-being	Communities that foster the abilities of older people
13	Lallukka et al.^19^	Joint trajectories of physical activity, health, and income before and after statutory retirement: a 22-year follow-up	Physical activity, health, and income	Communities that foster the abilities of older people
14	Yoneda et al.^20^	The importance of engaging in physical activity in older adulthood for transitions between cognitive status categories and death: a coordinated analysis of 14 longitudinal studies	Physical activity and cognition	Person-centered integrated care
15	Tiraphat et al.^21^	Active aging in ASEAN countries: influences from age-friendly environments, lifestyles, and socio-demographic factors	Lifestyle and chronic diseases	Person-centered integrated care
16	Liu et al.^22^	Healthy lifestyles are associated with better vitamin D status in community-dwelling older men: the Health In Men Study (HIMS)	Physical activity and cognitive decline	Person-centered integrated care
17	Chu et al.^23^	Sedentary behavior, physical activity, social participation, and loneliness among community-dwelling older adults in China	Healthy lifestyle	Communities that foster the abilities of older people
18	Szychowska and Drygas^24^	Physical activity as a determinant of successful aging: a narrative review article.	Diet and cognitive stimulation	Communities that foster the abilities of older people
19	Tcymbal et al.^25^	Interventions simultaneously promoting social participation and physical activity in community living older adults: a systematic review	Physical activity and cognition	Person-centered integrated care
20	Hu and Feng^26^	Association between community-based health services and health lifestyles of older adults: evidence from China	Lifestyle and mental health	Person-centered integrated care
21	Xu et al.^27^	Intrinsic capacity and health-promoting lifestyle in older adults: a latent class analysis	Lifestyle-based intervention	Communities that foster the abilities of older people
22	Zainab et al.^28^	Healthy ageing: assessment of health-promoting lifestyle among the elderly population in Karachi, Pakistan	Lifestyle and cognition	Person-centered integrated care
23	Peterson et al.^29^	Healthy ageing in the far North: perspectives and prescriptions	Lifestyle factors	Person-centered integrated care
24	Jiang et al.^30^	Effects of personal and health characteristics on the intrinsic capacity of older adults in the community: a cross-sectional study using the healthy aging framework	Multidomain interventions	Person-centered integrated care
25	Snigdha et al.^31^	Yoga-based lifestyle intervention for healthy ageing in older adults: a twoarmed, waitlist randomized controlled trial with multiple primary outcomes	Health-promoting lifestyle	Communities that foster the abilities of older people

Source: Authors’ elaboration based on the studies included in the integrative review (2020–2025).

Although the included studies showed a convergence with the action areas of the Decade of Healthy Ageing, this correspondence was not methodologically uniform across domains. Evidence linked to communities that foster the abilities of older people and person-centered integrated care was more frequent and generally supported by stronger observational and longitudinal designs, whereas evidence related to combating ageism and broader structural dimensions was less frequent and methodologically more limited.

Most of the identified evidence was concentrated in the action areas related to developing communities that foster the abilities of older people and to the provision of person-centered integrated care. To a lesser extent, some studies also addressed aspects related to combating ageism, particularly when discussing quality of life, autonomy, and dignity in older age.

In addition to the thematic analysis of the results, the studies were also classified according to methodological design in order to identify the predominant types of research used in the recent literature on healthy ageing. Table 4 presents the distribution of the included studies according to methodological design.

**Table 4:** Distribution of the included studies according to methodological design.

Methodological design	n	%
Cross-sectional studies	10	40
Longitudinal/cohort studies	5	20
Clinical trials/intervention studies	3	12
Qualitative studies	3	12
Literature reviews/narrative reviews	3	12
Pilot study	1	4
Total	25	100

Source: Authors’ elaboration based on the studies included in the integrative review.

The predominance of observational studies, especially cross-sectional designs, indicates that the current evidence base is stronger for identifying associations than for establishing causal relationships. In contrast, longitudinal studies and randomized controlled trials, although fewer in number, provide more robust support for temporal and interventional inferences.

A predominance of observational designs was identified, especially cross-sectional studies, which accounted for 40% of the included publications. Longitudinal or cohort studies represented 20% of the sample, while clinical trials or intervention studies, qualitative studies, and literature reviews each accounted for 12%, and one pilot study represented 4%. This distribution indicates that the current literature on healthy ageing is still largely based on observational evidence, with fewer intervention-based studies capable of supporting stronger causal inference.

This distribution indicates that the scientific production on healthy ageing has been predominantly conducted through observational research designs, with a smaller proportion of experimental or intervention-based investigations.

It is noteworthy that the analytical pillars discussed in this study were not originally developed by the authors. These dimensions emerged from the thematic analysis of the included studies and were interpreted in light of the guidelines established by the Decade of Healthy Ageing (2021–2030), a global initiative coordinated by the WHO that proposes strategic actions to promote health, functionality, and quality of life among older adults.

After completing the screening and eligibility process, 25 studies were included in the final synthesis. These publications represent diverse research designs and methodological approaches that address various aspects of healthy and active aging within the framework of the Decade of Healthy Aging (2021–2030).

## DISCUSSION

The present review identified dimensions associated with active ageing within the framework of the Decade of Healthy Ageing (2021–2030), based on evidence from different countries and sociocultural settings. Overall, the findings indicate that healthy ageing is a multidimensional process influenced by behavioral, social, economic, cultural, and symbolic factors. These findings suggest not only a multidimensional model of healthy ageing, but also an imbalance in the way ageing is conceptualized in the literature, with a predominance of studies centered on individual behavioral factors. This interpretation is consistent with the proposal of the Decade of Healthy Ageing, which emphasizes functional ability, supportive environments, and person-centered responses to ageing-related needs^1,2^.

A first aspect that stands out in the analyzed literature is the predominance of studies focused on modifiable lifestyle-related factors, particularly physical activity and diet. This concentration suggests that the current scientific literature on healthy ageing has prioritized individual behavioral determinants as central elements for the promotion of health in later life. Across the included studies, regular physical activity was repeatedly associated with maintenance of functional capacity, lower mortality risk, and better cognitive outcomes in older age^20^. Likewise, healthy dietary patterns were associated with reduced risk of chronic diseases and more favorable cognitive trajectories, especially in populations at increased risk of dementia^11,16^.

The importance attributed to lifestyle-related factors is also reinforced by studies showing that these effects are not limited to physical outcomes. Dietary patterns and cognitively stimulating lifestyle behaviors were associated with better cognitive performance and more favorable cognitive trajectories^11^, while broader healthy lifestyle profiles were related to longer life expectancy without Alzheimer’s dementia and to multidimensional indicators of successful aging^14,15^. In addition, lifestyle-oriented interventions and digital health strategies have shown potential to improve physical activity, dietary behaviors, and cardiovascular risk factors among older adults^10^. This finding suggests that active ageing should not be understood merely as the absence of disease, but as the result of sustained behaviors that support autonomy, functionality, and quality of life over the life course.

In addition to lifestyle, the review identified social engagement and cognitive stimulation as relevant dimensions of active ageing. Evidence indicates that education, occupational complexity, and participation in social activities are components of a cognitively stimulating lifestyle and may contribute to better cognitive performance in older adults^11^. Other studies also reinforce the relationship between health behaviors, multidimensional health, life satisfaction, and cognitive outcomes in later life^18,20^. In this sense, the literature supports the interpretation that healthy ageing involves not only biomedical protection, but also continued participation in meaningful social and intellectual activities. This is especially relevant in the context of the Decade of Healthy Ageing, since community participation and supportive environments are key elements for maintaining ability in later life^1,2^.

Another important finding concerns the role of socioeconomic determinants. Although fewer studies addressed this dimension directly, the available evidence suggests that income, access to resources, age-friendly environments, and favorable living conditions are associated with healthier ageing trajectories and greater adherence to health-promoting behaviors^19,21^. Conversely, social inequalities may increase vulnerability and restrict opportunities for active ageing. This indicates that healthy ageing cannot be reduced to individual responsibility alone, since structural conditions shape both the opportunities and constraints that older adults experience throughout the ageing process.

The review also identified spirituality and subjective well-being as relevant, although less frequently explored, dimensions. Evidence from qualitative research suggests that spirituality may support health maintenance by strengthening resilience, meaning in life, social connection, and subjective well-being, especially in contexts marked by migration and cultural adaptation^8^. At the same time, the importance attributed to spirituality appears to vary according to cultural context, which limits broad generalization and suggests that this dimension should be interpreted with cultural sensitivity. Rather than a universal predictor, spirituality may function as a contextual resource that becomes more salient in specific populations.

A further emerging dimension was the use of digital health technologies. The evidence available in the review suggests that digital tools, such as monitoring applications, wearable devices, and personalized interventions, may encourage healthy eating, physical activity, and cardiovascular risk reduction among older adults^10^. However, this body of evidence remains limited and concentrated in specific contexts, indicating that the role of digital health in active ageing is promising but still insufficiently explored. Issues such as accessibility, usability, digital literacy, and adherence should therefore be examined in more depth in future studies.

Taken together, the findings suggest that the five pillars identified in this review do not act independently. Instead, they seem to interact across individual, relational, and structural levels. Lifestyle behaviors are influenced by social participation, material living conditions, access to services, and, in some contexts, by symbolic dimensions such as spirituality. This integrative perspective is particularly relevant because it helps move the debate beyond narrow individual-centered approaches and highlights the complexity of healthy ageing as a socially embedded process.

These findings can also be more fully interpreted through the perspective of the life-course approach and the framework of the social determinants of health. From a life-course perspective, healthy ageing cannot be understood as the simple result of isolated behaviors adopted in later life, but rather as the cumulative product of exposures, opportunities, constraints, and social experiences that unfold over time. Likewise, the framework of the social determinants of health reinforces that income, education, living conditions, access to services, and social protection are not secondary background factors, but central conditions that shape the possibilities for autonomy, participation, and well-being in older age.

Although the dimensions identified in this review are broadly aligned with the action areas proposed by the WHO, important gaps remain in the translation of evidence into structural interventions and public policies. Much of the available literature emphasizes individual adaptation and lifestyle modification, while giving less attention to the institutional, community, and policy-level changes required to reduce inequalities and support healthy ageing in a more equitable way. This gap suggests that alignment with the WHO framework is conceptually relevant, but still insufficiently operationalized in terms of structural implementation.

At the same time, the review revealed important limitations in the current literature. Most included studies used observational designs, particularly cross-sectional methods, which are useful for identifying associations but do not allow robust causal inference. In addition, the methodological heterogeneity of the studies, including different populations, measures, and analytical strategies, limits direct comparison across findings. These aspects indicate that caution is needed when interpreting the evidence and suggest the need for more longitudinal and intervention-based studies to better clarify the mechanisms that support active and healthy ageing.

Another relevant point is that the distribution of evidence across dimensions was not homogeneous. Most studies concentrated on lifestyle-related behaviors, whereas structural and cultural dimensions, such as socioeconomic determinants and spirituality, appeared less frequently. This imbalance suggests that the scientific field still privileges modifiable individual behaviors over broader contextual explanations. Future research should therefore expand the investigation of how social inequalities, community environments, public services, and cultural meanings interact with behavioral factors in shaping ageing trajectories.

In summary, the literature analyzed in this review supports the understanding of active ageing as a multidimensional phenomenon in which healthy behaviors, social and cognitive engagement, material conditions, spirituality, and technological resources may all play a role. Rather than isolated domains, these dimensions should be interpreted as interconnected components of ageing processes that unfold across the life course. This perspective may contribute to the development of more comprehensive strategies capable of supporting autonomy, participation, and quality of life in older age.

### Study limitations and risk of bias

Although this integrative review was conducted in a systematic and transparent manner, some limitations should be considered when interpreting the results. First, a potential selection bias should be noted, as only studies available in full text were included. In addition, the use of text availability or open-access filters in some databases may have introduced selection bias by excluding potentially relevant studies not freely available in full text. A possible publication bias should also be considered, since studies with positive or statistically significant results tend to be published more frequently than studies reporting neutral or negative findings. Furthermore, the inclusion of articles only in Portuguese, English, and Spanish may have limited the scope of the review, representing a potential language bias. Although a formal methodological quality assessment was conducted using the JBI Critical Appraisal Tools, the heterogeneity of study designs, populations, measures, and analytical strategies still limited direct comparability across studies and reduced the strength of causal interpretation.

Finally, the possibility of interpretative bias in the categorization of findings according to the framework of the Decade of Healthy Ageing is acknowledged. However, efforts were made to minimize potential biases through independent screening of the studies by two reviewers, the use of the Rayyan platform for reference management, the application of previously defined eligibility criteria, and the formal appraisal of methodological quality.

In conclusion, the present review showed that the pillars associated with active ageing include healthy lifestyle behaviors, social and cognitive engagement, spirituality, socioeconomic determinants, and the use of digital health technologies. The findings indicate that healthy ageing is a multidimensional process that goes beyond the absence of disease, involving the maintenance of functional capacity, autonomy, and quality of life throughout the life course. Although the findings reinforce the multidimensional nature of healthy ageing, the current evidence base remains disproportionately focused on individual behaviors, with limited attention to structural determinants such as inequality, access to services, and social protection.

Within the context of the Decade of Healthy Ageing (2021–2030), these results reinforce the importance of public policies and interventions that integrate health promotion actions, of strengthening social networks, and guaranteeing favorable social conditions for ageing. Thus, promoting active ageing requires intersectoral, culturally sensitive, and person-centered strategies that articulate individual, social, and structural factors in order to sustain autonomy, participation, and quality of life in older age. Future research should prioritize longitudinal and intervention-based designs capable of clarifying causal pathways and generating evidence that is more robust for policy and practice.

## Data Availability

The datasets generated and/or analyzed during the current study are available from the corresponding author upon reasonable request.

## References

[1] 1. World Health Organization. Decade of healthy ageing: baseline report [Internet]. Geneva: World Health Organization; 2020 [cited on Nov 11, 2205]. Available from: https://iris.who.int/bitstream/handle/10665/338677/9789240017900-eng.pdf

[2] 2. Pan American Health Organization. The decade of healthy ageing in the Americas (2021–2030) [Internet]. Washington: Pan American Health Organization; 2021 [cited on Nov 11, 2205]. Available from: https://www.paho.org/en/decade-healthy-aging-americas-2021-2030

[3] 3. Souza MT, Silva MD, Carvalho R. Revisão integrativa: o que é e como fazer. Einstein. 2010;8(1):102-6. https://doi.org/10.1590/S1679-45082010RW1134 10.1590/S1679-45082010RW1134 26761761

[4] 4. Peters MDJ, Godfrey CM, Khalil H, McInerney P, Parker D, Soares CB. Guidance for conducting systematic scoping reviews. Int J Evid Based Healthc. 2015;13(3):141-6. https://doi.org/10.1097/XEB.0000000000000050 10.1097/XEB.0000000000000050 26134548

[5] 5. Silva MS, Silva ALG, Silva FHAF, Luz MCNP, Nunes VMA. Pilares para a velhice ativa na década do envelhecimento saudável: revisão integrativa da literatura. Open Science Framework; 2025. https://doi.org/10.17605/OSF.IO/AM3HB

[6] 6. Page MJ, McKenzie JE, Bossuyt PM, Boutron I, Hoffmann TC, Mulrow CD, et al. The PRISMA 2020 statement: an updated guideline for reporting systematic reviews. BMJ. 2021;372:n71. https://doi.org/10.1136/bmj.n71 10.1136/bmj.n71 PMC8005924 33782057

[7] 7. Alali DS, Alshebly AA, Alajlani A, Al Jumaiei AH, Alghadeer ZM, Ali SI. Awareness of healthy lifestyle among elderly population during aging in Al-Ahsa, Saudi Arabia. Cureus. 2023;15(11):e49054. https://doi.org/10.7759/cureus.49054 10.7759/cureus.49054 PMC10731629 38125212

[8] 8. Weiner-Light S, Rankin KP, Lanata S, Possin KL, Dohan D, Sideman AB. The role of spirituality in conceptualizations of health maintenance and healthy aging among Latin American immigrants. Am J Geriatr Psychiatry. 2021;29(11):1079-88. https://doi.org/10.1016/j.jagp.2021.04.017 10.1016/j.jagp.2021.04.017 PMC8846417 34092458

[9] 9. Andreo-López MC, Contreras-Bolívar V, Muñoz-Torres M, García-Fontana B, García-Fontana C. Influence of the Mediterranean diet on healthy aging. Int J Mol Sci. 2023;24(5):4491. https://doi.org/10.3390/ijms24054491 10.3390/ijms24054491 PMC10003249 36901921

[10] 10. Cacciata M, Candelaria D, Reyes AT, Serafica R, Hildebrand JA, Santa Maria A, et al. Digital health technologies to promote healthy eating and physical activity and reduce risk factors for cardiovascular disease in older adults: a pilot study. J Cardiovasc Nurs. 2025;40(5):475-85. https://doi.org/10.1097/JCN.0000000000001184 10.1097/JCN.0000000000001184 PMC12334334 40059110

[11] 11. Parrott MD, Carmichael PH, Laurin D, Greenwood CE, Anderson ND, Ferland G, et al. The association between dietary pattern adherence, cognitive stimulating lifestyle, and cognitive function among older adults from the Quebec Longitudinal Study on Nutrition and Successful Aging. J Gerontol B Psychol Sci Soc Sci. 2021;76(3):444-50. https://doi.org/10.1093/geronb/gbaa178 10.1093/geronb/gbaa178 PMC7887726 33063101

[12] 12. Eymundsdottir H, Chang M, Geirsdottir OG, Gunnarsdottir I, Jonsson PV, Gudnason V, et al. Lifestyle and 25-hydroxy-vitamin D among community-dwelling old adults with dementia, mild cognitive impairment, or normal cognitive function. Aging Clin Exp Res. 2020;32(12):2649-56. https://doi.org/10.1007/s40520-020-01531-1 10.1007/s40520-020-01531-1 PMC9233863 32248358

[13] 13. Martin P, Poon LW; Georgia Centenarian Study. Healthy aging among centenarians and near-centenarians: findings from the Georgia Centenarian Study. Maturitas. 2024;185:108001. https://doi.org/10.1016/j.maturitas.2024.108001 10.1016/j.maturitas.2024.108001 PMC11219216 38677175

[14] 14. Dhana K, Franco OH, Ritz EM, Ford CN, Desai P, Krueger KR, et al. Healthy lifestyle and life expectancy with and without Alzheimer’s dementia: population based cohort study. BMJ. 2022;377:e068390. https://doi.org/10.1136/bmj-2021-068390 10.1136/bmj-2021-068390 PMC9006322 35418416

[15] 15. Lee-Bravatti MA, O’Neill HJ, Wurth RC, Sotos-Prieto M, Gao X, Falcon LM, et al. Lifestyle behavioral factors and integrative successful aging among Puerto Ricans living in the mainland United States. J Gerontol A Biol Sci Med Sci. 2021;76(6):1108-16. https://doi.org/10.1093/gerona/glaa259 10.1093/gerona/glaa259 PMC8248899 33045072

[16] 16. Blotenberg I, Zülke AE, Luppa M, Wittmann F, Fankhänel T, Weise S, et al. Factors associated with a healthy diet and willingness to change dietary behavior in older adults at increased risk of dementia. J Alzheimers Dis. 2025;105(2):634-45. https://doi.org/10.1177/13872877251330296 10.1177/13872877251330296 PMC12231830 40232259

[17] 17. Zadworna M. Pathways to healthy aging: exploring the determinants of self-rated health in older adults. Acta Psychol (Amst). 2022;228:103651. https://doi.org/10.1016/j.actpsy.2022.103651 10.1016/j.actpsy.2022.103651 35785683

[18] 18. Zhang J, Zhang Y, Wu Z, Fu X. Enhancing understanding of healthy aging based on time-varying dependencies among multidimensional health, life satisfaction, and health behaviors of older adults aged 60 years and over. BMC Public Health. 2024;24(1):192. https://doi.org/10.1186/s12889-024-17752-2 10.1186/s12889-024-17752-2 PMC10790531 38229050

[19] 19. Lallukka T, Kolmonen P, Rahkonen O, Lahelma E, Lahti J. Joint trajectories of physical activity, health, and income before and after statutory retirement: a 22-year follow-up. PLoS One. 2025;20(1):e0317010. https://doi.org/10.1371/journal.pone.0317010 10.1371/journal.pone.0317010 PMC11778762 39879149

[20] 20. Yoneda T, Lewis NA, Knight JE, Rush J, Vendittelli R, Kleineidam L, et al. The importance of engaging in physical activity in older adulthood for transitions between cognitive status categories and death: a coordinated analysis of 14 longitudinal studies. J Gerontol A Biol Sci Med Sci. 2021;76(9):1668-77. https://doi.org/10.1093/gerona/glaa268 10.1093/gerona/glaa268 PMC8361336 33099603

[21] 21. Tiraphat S, Kasemsup V, Buntup D, Munisamy M, Nguyen TH, Myint AH. Active aging in ASEAN countries: influences from age-friendly environments, lifestyles, and socio-demographic factors. Int J Environ Res Public Health. 2021;18(16):8290. https://doi.org/10.3390/ijerph18168290 10.3390/ijerph18168290 PMC8391192 34444040

[22] 22. Liu X, Brock KE, Brennan-Speranza TC, Flicker L, Golledge J, Hankey GJ, et al. Healthy lifestyles are associated with better vitamin D status in community-dwelling older men: the Health In Men Study (HIMS). Clin Endocrinol (Oxf). 2023;99(2):165-73. https://doi.org/10.1111/cen.14926 10.1111/cen.14926 PMC10952998 37165475

[23] 23. Chu A, Lu Y, Zhang H, Jiang Y. Sedentary behavior, physical activity, social participation, and loneliness among community-dwelling older adults in China. J Aging Phys Act. 2023;31(6):987-94. https://doi.org/10.1123/japa.2022-0205 10.1123/japa.2022-0205 37442551

[24] 24. Szychowska A, Drygas W. Physical activity as a determinant of successful aging: a narrative review article. Aging Clin Exp Res. 2022;34(6):1209-14. https://doi.org/10.1007/s40520-021-02037-0 10.1007/s40520-021-02037-0 PMC9151514 34873677

[25] 25. Tcymbal A, Abu-Omar K, Hartung V, Bußkamp A, Comito C, Rossmann C, et al. Interventions simultaneously promoting social participation and physical activity in community living older adults: a systematic review. Front Public Health. 2022;10:1048496. https://doi.org/10.3389/fpubh.2022.1048496 10.3389/fpubh.2022.1048496 PMC9768837 36568739

[26] 26. Hu Y, Feng T. Association between community-based health services and health lifestyles of older adults: evidence from China. BMC Geriatr. 2025;25(1):450. https://doi.org/10.1186/s12877-025-06119-w 10.1186/s12877-025-06119-w PMC12220610 40604431

[27] 27. Xu C, Yu J, Yang L, Li Y, Ma D. Intrinsic capacity and health-promoting lifestyle in older adults: a latent class analysis. Front Public Health. 2025;13:1634373. https://doi.org/10.3389/fpubh.2025.1634373 10.3389/fpubh.2025.1634373 PMC12307196 40740367

[28] 28. Zainab S, Khoso A, Siddiqui M, Ashraf K, Mumtaz MA, Awan M. Healthy ageing: assessment of health-promoting lifestyle among the elderly population in Karachi, Pakistan. J Educ Health Promot. 2021;10:389. https://doi.org/10.4103/jehp.jehp_241_21 10.4103/jehp.jehp_241_21 PMC8641717 34912925

[29] 29. Peterson JR, Baumgartner DA, Austin SL. Healthy ageing in the far North: perspectives and prescriptions. Int J Circumpolar Health. 2020;79(1):1735036. https://doi.org/10.1080/22423982.2020.1735036 10.1080/22423982.2020.1735036 PMC7067180 32114971

[30] 30. Jiang X, Chen F, Yang X, Yang M, Zhang X, Ma X, et al. Effects of personal and health characteristics on the intrinsic capacity of older adults in the community: a cross-sectional study using the healthy aging framework. BMC Geriatr. 2023;23(1):643. https://doi.org/10.1186/s12877-02304362-7 10.1186/s12877-023-04362-7 PMC10566030 37817083

[31] 31. Snigdha A, Majumdar V, Manjunath NK, Jose A. Yoga-based lifestyle intervention for healthy ageing in older adults: a two-armed, waitlist randomized controlled trial with multiple primary outcomes. Geroscience. 2024;46(6):6039-54. https://doi.org/10.1007/s11357-024-01149-5 10.1007/s11357-024-01149-5 PMC11493921 38583114

